# One Health alert: zoonotic scabies from dromedary camels—A case report and call for vigilance

**DOI:** 10.3389/fvets.2025.1500916

**Published:** 2025-03-12

**Authors:** Hebel Christiana, Schuster Rolf Karl, Kinne Joerg, Wernery Ulrich

**Affiliations:** Central Veterinary Research Laboratory, Dubai, United Arab Emirates

**Keywords:** dromedary camel, sarcoptes scabiei, sarcoptic mange, pseudoscabies, zoonosis, One Health

## Abstract

This case report describes the rapid transmission of sarcoptic mange, a highly contagious skin disease caused by the mite *Sarcoptes scabiei*, from one dromedary camel to a group of other dromedary camels and a group of human beings despite wearing personal protective equipment. This is the first report on an outbreak of human pseudoscabies in the United Arab Emirates, and it highlights the importance of a One Health approach managing zoonotic diseases. Early detection, treatment of infected animals, as well as adherence to hygiene and quarantine protocols are crucial to prevent zoonotic spillovers.

## 1 Introduction

Dromedary camels (*Camelus dromedarius*) (following referred to as camel) are not only an important part of the cultural heritage in the Middle East by being Bedouin companions, providing transportation, milk and wool, but they also play a role in modern life as they are still bred for milk and meat, as well as for prestigious camel races (1). The bond between camels and their owners and caretakers is strong, and close contact is very common. This provides not only a lot of benefits, but does also carry the risk of disease transmission (2). Possible zoonotic transmission of different parasites from camels to human beings are described (3). One of them is sarcoptic mange, a highly contagious skin disease caused by the mite *Sarcoptes scabiei* (4). It affects various mammals, including humans, camels, and other livestock. While interspecies transmission of sarcoptic mange is common, documented cases of camel-to-human transmission are rare, and reported to occur mainly during milking (5). Transferred to humans, the sarcoptic mange is a highly contagious skin disease called “Pseudoscabies” causing a temporary infestation with mites. Unlike true scabies, which is caused in human beings by *S. scabiei* var. *hominis* and listed by the World Health Organization (WHO) as a neglected tropical disease in 2017, and which is highly contagious between humans, pseudoscabies is very rarely transmittable from person to person (6).

This case report presents the rapid transmission of sarcoptic mange from camels to a group of human beings, despite wearing personal protective equipment, highlighting the importance of a One Health approach in managing zoonotic diseases.

## 2 Case description

### 2.1 Case presentation of the camel

**Figure 1:**
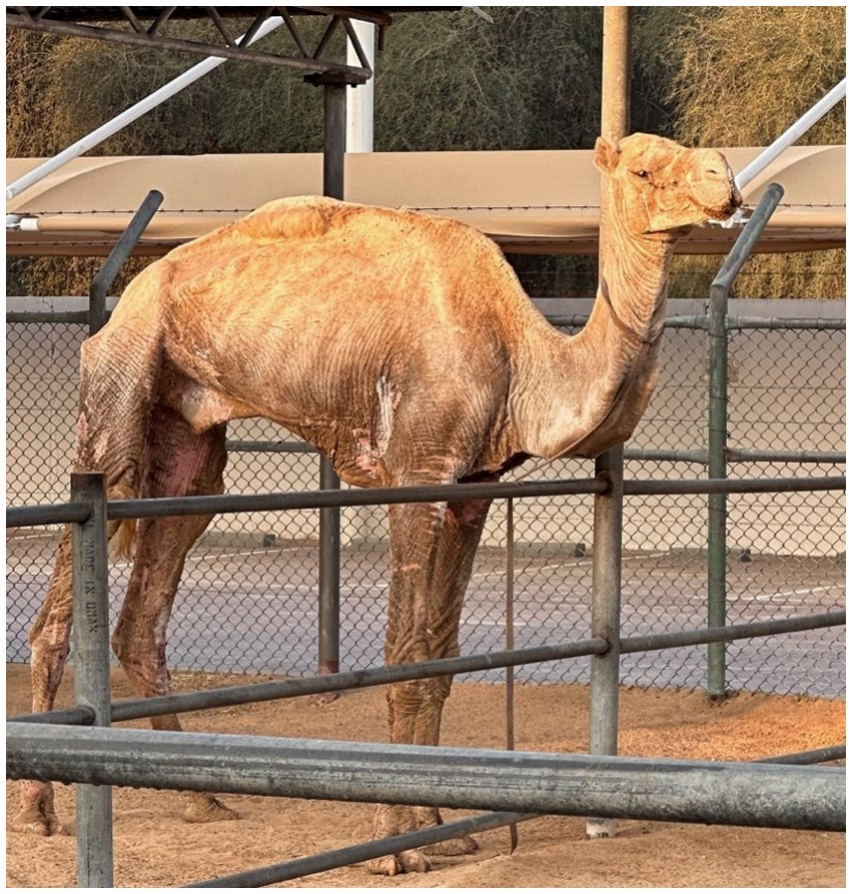
Index case: Camel with severe alopecia and hyperkeratosis caused by *Sarcoptes scabiei* infestation.

A 2-year old castrated, male camel was presented with a month-long history of hyperkeratosis, severe pruritus, alopecia, anorexia and dark brown color change of its skin all over the body. Several areas of the body were covered with one-centimeter-thick crusts and severely thickened involving the ears, nostrils, neck, and lower limbs (Figure 1), with several areas exhibiting half centimeter deep cracks infested with maggots of the species *Wohlfahrtia nuba*. In addition, the camel suffered from a tick (*Hyalomma dromedarii*) infestation. The camel was housed at a private farm with 19 other camels, that presented similar skin lesions of different severity. All animals were allowed to roam around the desert freely during the day.

The camel was moved to a treatment facility in Dubai, United Arab Emirates (UAE), that kept 23 camels (in groups of 3 or 4 camels), 2 donkeys and 12 sheep. Blood collection on arrival revealed an increased total white blood cell count (18.5 × 10^9^/L), with neutrophils and eosinophils increased, a PCV of 0.21 L/L and slightly lowered iron 10 μmoL/L. Biochemistry results showed elevated CK (262 U/L) and LDH (584 U/L), while other values were within normal limits.

The camel was maintained in isolation, segregated from other animals. The only interaction occurred on the arrival day, when two resident camels had short contact by sniffing on it when it first walked into the facility. The treatment facility holding areas are simple pens with sand paddocks with iron fencing. Food troughs are made of steel. On arrival, five different people wearing Nitril gloves and working overalls handled the camel.

**Figure 2:**
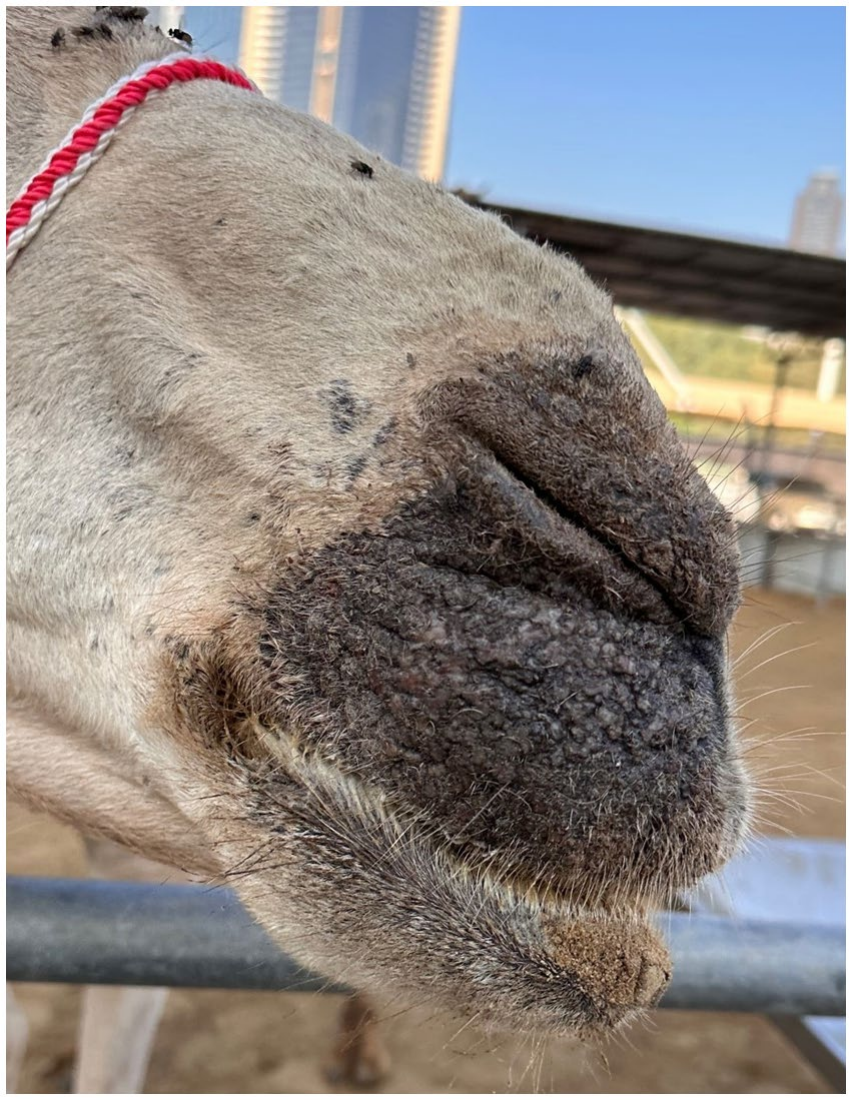
Contact animal: Alopecia and hyperkeratosis caused by *Sarcoptes scabiei* infestation starting around the nostrils.

Within three days after the primary interaction at the treatment facility, two of the resident camels, that had less than a minute contact by only sniffing on day one, started showing typical mange lesions around the nostrils and lips (Figure 2). The camels did not share the same feeding troughs. Skin scrapings from these two camels revealed a *S. scabiei* infestation.

### 2.2 Case presentation of the workers

**Figure 3:**
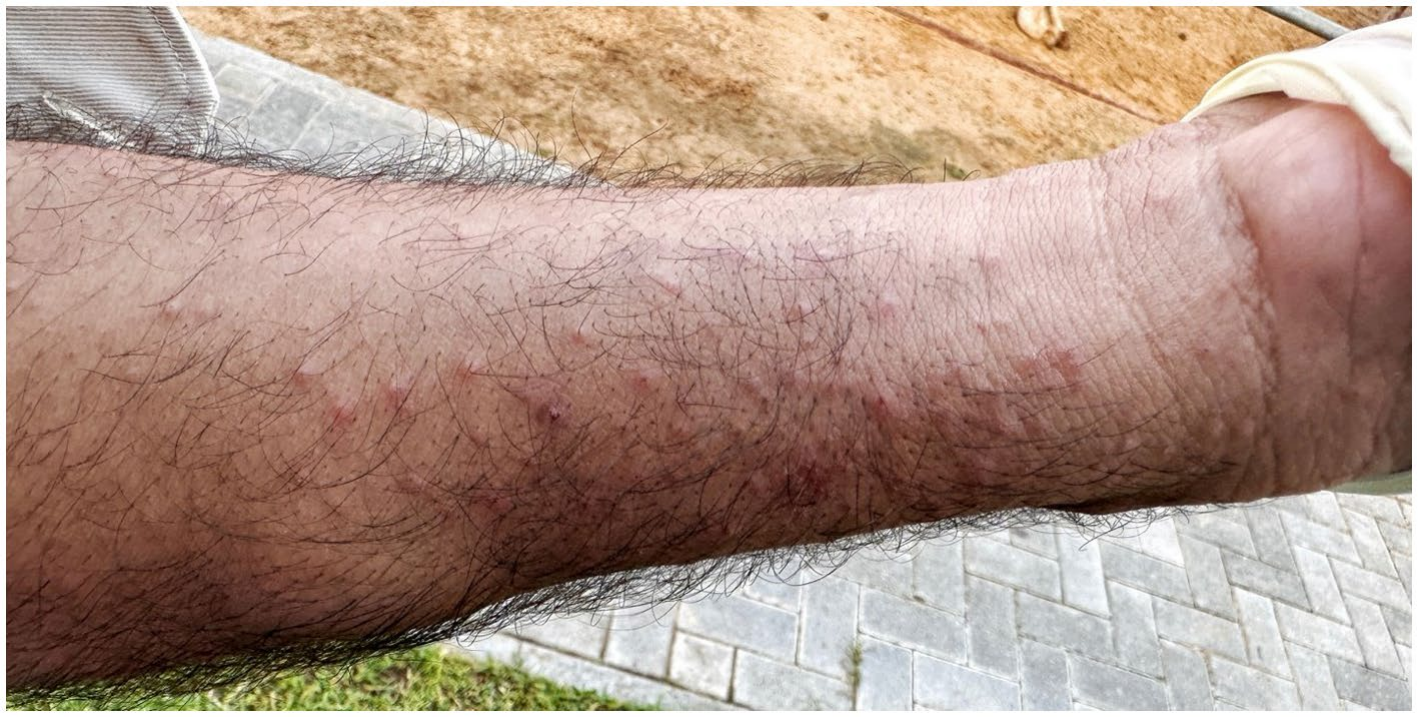
Skin lesions of the forearm of the camel caretaker showing several erythematous papules and pustules.

Despite wearing nitril gloves on the first day and changing their working overalls, all workers handling the camel developed intensely pruritic erythematous papules and pustules on their hands, wrists, forearms, thorax, abdomen and thighs within three days of handling the index camel. The caretakers had contact with the camel while feeding and cleaning its enclosure and treatment. Upon examination of the caretakers’ skin, burrows characteristic of scabies mite infestation was identified on the affected areas. A sixth and seventh person who only started handling the camel on day four and five after arrival, also showed pruritus and erythematous papules three days after the first contact (Figure 3).

Upon investigation, the camel handlers from the original farm also confirmed that they had severe pruritus and erythematous papules at several areas of the body.

## 3 Management and control

The newly arrived camel was treated with a dose of 0.2 mg/kg ivermectin injection (Ivomec, Merial, details 1 mg/mL) on day one of its arrival. In addition, it received flumethrin (Bayticol, Bayer) against ticks. Furthermore, besides the antiparasitic treatment, the index camel’s cracked wounds were washed with chlorhexidine 3% and the maggots were flushed out with hydrogen peroxide. Afterwards, the skin was oiled with 100% natural coconut oil to soften the crusts, and permethrin antifly repellent spray was applied topically. In addition, it received treatment with 5 mg/kg enrofloxacin (Baytril 10%, Bayer, Germany) intravenously for 5 days for secondary infections and a single dose with 0.6 mg/kg meloxicam (Metacam, Boehringer Ingelheim, Germany). Water was given orally by pouring it into the mouth, which the camel swallowed. It occasionally did take carrots and dates by hand. On day 5 the camel still showed anorexia, severe pruritus and apathy. Repeated skin scrapings taken still showed live mites. Therefore, the camel was then treated with doramectin (Dectomax, Zoetis, Egypt) injection 0.2 mg/kg subcutaneous. The camel was found dead on day 8 at the treatment facility. Postmortem examination revealed emaciation, severe anemia, alopecia and severe hyperkeratosis. All internal organs were macroscopically unremarkable. Histopathological examination of skin samples from different areas showed a marked hyperkeratotic dermatitis with perivascular inflammation including eosinophils and cocci-shaped bacteria. *Sarcoptes* mites were seen in burrows of the stratum corneum layers of the skin. Additionally, the intestine showed a marked subacute eosinophilic enteritis with severe coccidiosis.

All other 17 camels at the original private farm, as well as all 23 camels a the treatment facility and the 12 sheep, were treated thrice with subcutaneous doramectin injections of 0.2 mg/kg of body weight every two weeks and once with pour-on ivermectin (5 mg/mL Ivermectin, Durvet, Inc. Bluesprings, Missouri, USA) poured onto the hump area. Repeated skin scrapings from the infected animals showed significant reduction of live mites within 48 h from the first treatment. Nevertheless, 2 months after the third doramectin injection, several camels at the treatment facility started showing thickened skin and severe pruritus at different areas of the body. Skin scrapings confirmed again *Sarcoptes* mites. At this point it was decided to go for a longer treatment regime with doramectin injections of 0.2 mg/kg every two weeks for two months.

Given the composition of the enclosures, consisting primarily of sand paddocks, iron fencing and steel troughs, disinfection procedures were not implemented, and would have been very difficult. Ropes and halters of the camels were replaced with new ones after the treatment.

The handlers received topical scabicidal treatment (permethrin 5%) and symptomatic relief for itching. However, the infected handlers kept showing symptoms of the pruritic erythematous papules and pustules as well as severe pruritus up to four weeks after the first encounter. New single papules and pustules showed up occasionally in different areas of the body for two more weeks.

## 4 Diagnostic assessment

**Figure 4:**
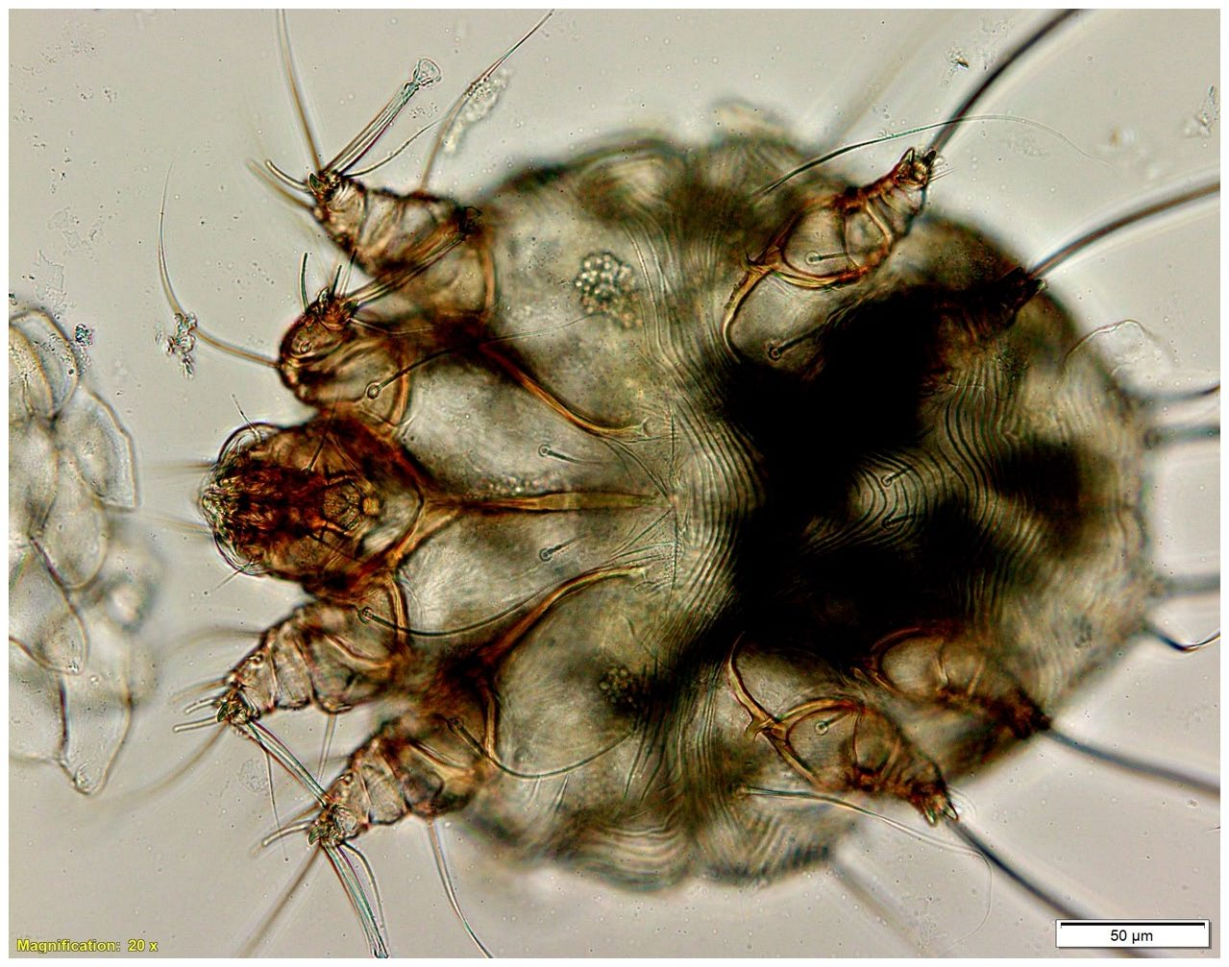
*Sarcoptes scabiei* mites 50 μm in size under the microscope 20x magnification.

Deep skin scrapings from the live camel containing crusts and scabs from seven different affected areas were collected and placed on filter paper discs in petri dishes for 24 h at 38°C (7). Consequently, after only one hour a large number of mites of different development stages moved out from all samples, crawling at the base of the petri dishes. The mites were identified as *S. scabiei*. (Figure 4).

Skin scrapings collected from the 19 other camels from the private farm displaying similar skin lesions like alopecia and hyperkeratosis as well as severe pruritus, tested positive for *Sarcoptes* mites.

## 5 Discussion

Sarcoptic mange is a highly contagious skin disease caused by the mite *S. scabiei*. It affects various domestic and a large variety of wild mammals and is by now found in more than 140 host species (8). In the UAE, the disease was recently reported in free-roaming Arabian Oryx (*Oryx leucoryx*) (9). Direct contact between animals is the primary transmission route, and mange in camels is extremely contagious (2).

Besides the very high morbidity, *S. scabiei* can also show high mortality in species like the Arabian Oryx (9). Treatment in camelids is described as challenging and reported to take a long time (10), the same was confirmed at the treatment facility where the mange flared up after discontinuing the treatment after the third injection of doramectin. Generalized sarcoptic mange leads to weight loss, abnormal behavior, increased susceptibility to other diseases, as well as increased mortality (11). Especially in young, old or immunocompromised animals it can lead to secondary bacterial infections, emaciation and death. The severe inflammation of the skin, the biggest organ of the body, reduces the natural immunological function. The affected animals in the original farm and at the treatment facility also showed restlessness and uneasiness whilst eating, due to the intense pruritus. As documented in this case report, the camel presented to the treatment facility exhibited the profound consequences of a severe mange infection: generalized weight loss, secondary bacterial infections, and an impaired immune system likely contributing to malabsorption due to coccidiosis.

Although camel mange caused by *S. scabiei* has been reported from various countries, like Nigeria (12), Ethiopia (24, 25), Egypt (13), Saudi Arabia (14), India (15) and Pakistan (16), there are currently no known case reports of sarcoptic mange outbreaks in camels in the UAE, even though the disease is very common.

In human beings the disease is caused by a host-specific *S. scabiei* strain and it is called scabies, which was listed by the WHO as a neglected tropical disease in 2017 (17). The contact of humans with mange infected animals can cause pseudoscabies also known as zoonotic scabies, a self-limiting condition that is characterized by severe pruritus. Moroni et al. (18) reviewed the presence of *S. scabiei* in various domestic and wild animals, including new world camelids, but did not list a documented case of a zoonotic outbreak from Dromedary or Bactrian camels. The development of pseudoscabies in humans after contact with infected camels is rare (2, 3, 5, 12). But as shown in this case, even a short contact with a severely infected animal, despite wearing personal protective equipment, is enough to get infected.

Due to the high zoonotic potential, sarcoptic mange in camels should be handled by a multifaceted approach. It is important to implement robust preventive measures, including quarantine protocols and regular inspections. Early detection and immediate treatment can possibly prevent severe mange outbreaks and zoonotic spillovers. Nevertheless, as shown in this case, wearing nitril gloves when handling animals infested with mange, did not guarantee protection, most likely due to the immense number of mites. Especially the crusted “Norwegian” from, which is linked to a high mite density, seems to harbor a higher infectious potential (18).

Infestations in human beings with pseudoscabies can be challenging to identify as they presnt differently than standard human scabies. Nonetheless, careful consideration of the patient’s history can lead to a definitive diagnosis (19). Especially because the distribution of the skin lesions was described as typical for zoonotic scabies (17). Infestations with pseudoscabies are usually self-limiting, but to speed up recovery up, topical treatment with 5% permethrin ointment should be considered (19).

For human scabies, different treatment protocols (oral ivermectin, repeated 1–2 weeks after) are available and treatment protocols for cattle and sheep are described in detail, but protocols for camels are based more on experience than scientific data (20–22). Doramectin in the dose of 0.2 mg/kg body weight subcutaneous biweekly for two month is the dose recommended to be used for treatment of scabies in dromedary camels (15), but pharmacological studies are missing.

This case highlights the One Health principle of interconnectedness between human, animal, and the environment. The close contact between camel handlers and infected animals, coupled with lax quarantine measures facilitated the zoonotic transmission of *S. scabiei* mites. It underscores the critical need for increased awareness among camel handlers, owners, veterinarians and public health officials, especially due to the close contact between camel handlers and their animals. It also demonstrates that even in routine animal husbandry practices, despite wearing personal protective equipment and changing clothes, the risk of transmission is still high.

Several environmental factors can contribute to the spread of sarcoptic mange in camels, including poor sanitation, overcrowding, and allowing camels to roam freely. Inadequate cleaning of stables and equipment allows mites to persist in the environment, while overcrowding in stables or during transport increases the frequency of direct contact between animals, facilitating the spread of mites. Free-roaming camels are also more likely to come into contact with infected animals. Additionally, climate and humidity can significantly influence the transmission and severity of sarcoptic mange, as these factors affect mite survival and reproduction rates. Sarcoptic mites can survive off the host for a limited time, typically a few weeks. Cool and humid conditions prolong their survival, while dry conditions at 37°C can limit their survival to less than 48 h based on our experience.

Sarcoptic mange is highly contagious and spreads easily within camel groups. Transmission primarily occurs through direct contact between infected animals, including horizontal transmission between animals within the same herd, vertical transmission from mother to offspring, and transmission between animals of different age groups. Indirect transmission can also occur through contaminated fomites such as bedding, grooming equipment, and ropes (23).

The crucial of responsible animal ownership needs to be addressed. Animal welfare is an often overlooked aspect when dealing with a severe mange infestation. Mange causes immense discomfort for the infected animals and predisposes the animals to secondary bacterial infections. Therefore, proper parasite control programmes are essential for the wellbeing of both animals and humans. Early detection and diagnosis of mange are crucial for prompt intervention and control, as evidenced by the rapid spread to the other camels and humans with just brief contact.

Veterinarians and public health officials must collaborate to implement effective prevention and control strategies. Educating the public, animal handlers and owners about the zoonotic potential of sarcoptic mange, including hygiene measures, prevention and treatment options, is paramount. The currently missing scientific data on optimal treatment of sarcoptic mange in camels needs further research.

In conclusion, this case report highlights the One Heath relevance of scabies. It underscores the need for integrated surveillance, prevention and control strategies to safeguard both human and animal health. Collaboration can effectively help managing zoonotic disease outbreaks. By fostering collaboration we can effectively manage zoonotic diseases and create a healthier environment for all.

## Data Availability

The raw data supporting the conclusions of this article will be made available by the authors, without undue reservation.
